# Supplementary material for: The causal relationship between the human gut microbiota and pyogenic arthritis: a Mendelian randomization study
Source: Front Cell Infect Microbiol. 2024 Nov 26;14:1452480. doi: 10.3389/fcimb.2024.1452480 (PMC11629706; doi:10.3389/fcimb.2024.1452480)
Supplement: Supplementary file 1 [file DataSheet1.zip › mendelian test/ebi-a-GCST90027573.csv_forest.pdf]

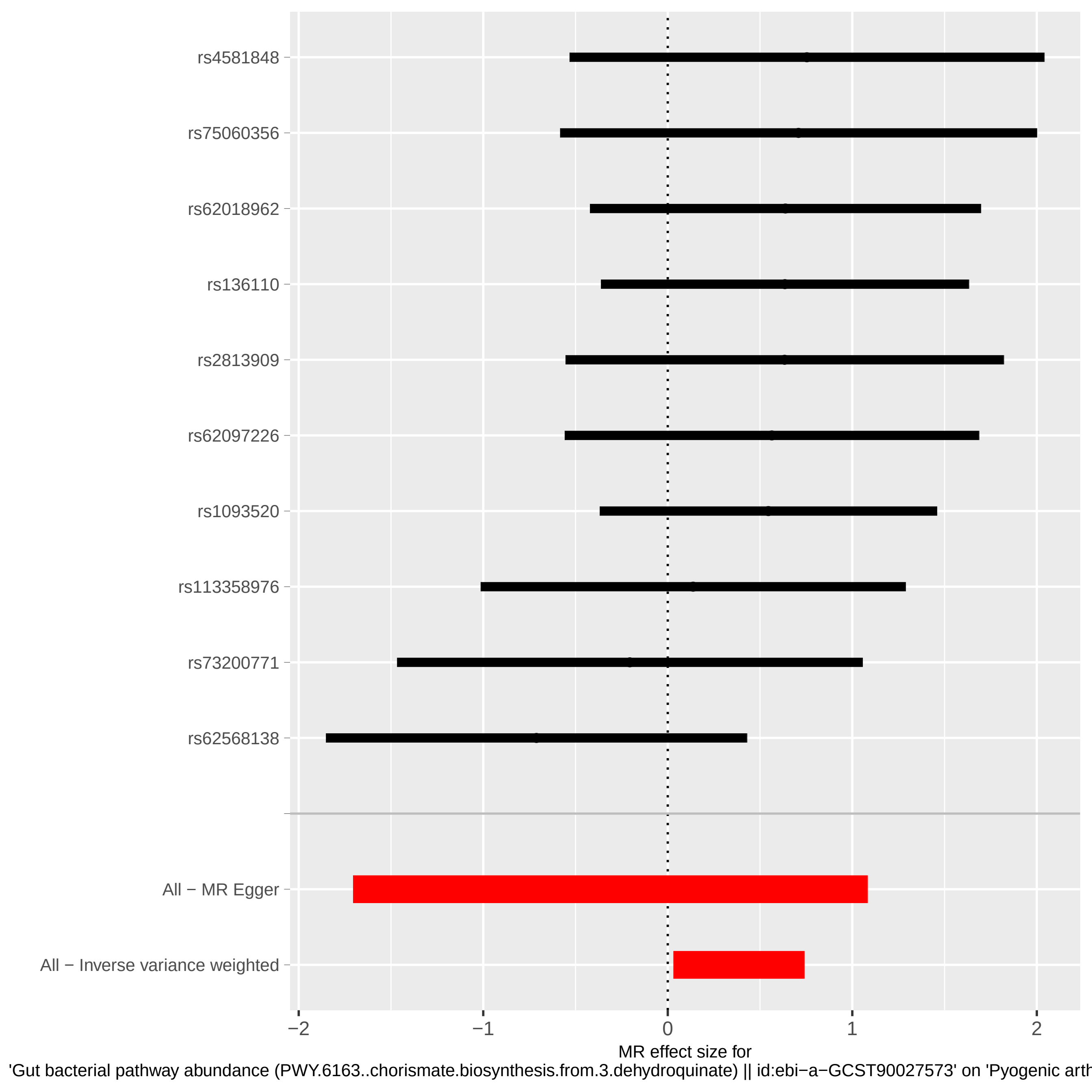
rs4581848
rs75060356
rs62018962
rs136110
rs2813909
rs62097226
rs1093520
rs113358976
rs73200771
rs62568138
All − MR Egger
All − Inverse variance weighted
−2
−1
0
1
2
MR effect size for
'Gut bacterial pathway abundance (PWY.6163..chorismate.biosynthesis.from.3.dehydroquinate) || id:ebi−a−GCST90027573' on 'Pyogenic arth
